# Supplementary material for: Recombinant Expression and Purification of the Cyanobacterial Chaperone HtpG from Synechococcus elongatus PCC 7942
Source: Methods Protoc. 2025 Sep 6;8(5):103. doi: 10.3390/mps8050103 (PMC12452654; doi:10.3390/mps8050103)
Supplement: Supplementary file 1 [file mps-08-00103-s001.zip › mps-3826706-supplementary.pdf]

Table S1 The list of strains used for sequence alignments

| **Abbreviation** | **Species** | **Class** | **Uniprot ID** |
|---|---|---|---|
| mtHtpG | *Mycobacterium tuberculosis* | Actinomycetes | P9WMJ7 |
| crHsp90 | *Chlamydomonas reinhardtii* | Chlorophyceae | A8J1U1 |
| aiHtpG | *Arthrospira innermongoliensis* BZ-2011 | Cyanophyceae | G3E1T1 |
| anHtpG | *Anabaena* sp. YBS01 | Cyanophyceae | A0A5Q0GLU6 |
| ftHtpG | *Fischerella thermalis* JSC-11 | Cyanophyceae | G6FZC6 |
| lmHtpG | *Limnospira maxima* CS-328 | Cyanophyceae | B5W034 |
| maHtpG | *Microcystis aeruginosa* PCC 9443 | Cyanophyceae | I4G8Q1 |
| npHtpG | *Nostoc punctiforme* NIES-2108 | Cyanophyceae | A0A367RKX7 |
| pmHtpG | *Prochlorococcus marinus* (strain AS9601) | Cyanophyceae | A2BR33 |
| **seHtpG** | *Synechococcus elongatus* PCC 7942 | Cyanophyceae | Q79N42 |
| spHtpG | *Spirulina* sp. SIO3F2 | Cyanophyceae | A0A845WVB2 |
| ssHtpG | *Synechococcus* sp. PCC 7002 | Cyanophyceae | B1XQJ4 |
| syHtpG | *Synechococcus* sp. (strain JA-3-3Ab) | Cyanophyceae | Q2JVG6 |
| ecHtpG | *Escherichia coli* (strain K12) | Gammaproteobacteria | P0A6Z3 |
| paHtpG | Pseudomonas aeruginosa | Gammaproteobacteria | Q9I3C5 |
| hsHsp90α | *Homo sapiens* | Mammalia | P07900 |
| hsHsp90β | *Homo sapiens* | Mammalia | P08238 |
| hsTrap1 | *Homo sapiens* | Mammalia | Q12931 |
| hsGrp94 | *Homo sapiens* | Mammalia | P14625 |
| atHsp901 | *Arabidopsis thaliana* | Rosids | P27323 |
| atHsp905 | *Arabidopsis thaliana* | Rosids | Q9SIF2 |
| scHsc82 | *Saccharomyces cerevisiae* | Saccharomycetes | P15108 |
| saHtpG | *Saccharolobus shibatae* | Thermoprotei | O05207 |
